# Comparative analysis of cytokine transcript profiles within mediastinal lymph node compartments of pigs after infection with porcine reproductive and respiratory syndrome genotype 1 strains differing in pathogenicity

**DOI:** 10.1186/s13567-015-0161-8

**Published:** 2015-03-19

**Authors:** Obdulio García-Nicolás, Rubén S Rosales, Francisco J Pallarés, David Risco, Juan J Quereda, Simon P Graham, Jean-Pierre Frossard, Sophie B Morgan, Falko Steinbach, Trevor W Drew, Tony S Strickland, Francisco J Salguero

**Affiliations:** Department of Anatomy and Comparative Pathology, Faculty of Veterinary Medicine, Murcia University, “Mare Nostrum Excellence Campus – 3738”, 30100 Murcia, Spain; Department of Bacteriology, Animal and Plant Health Agency, Addlestone, Surrey, KT15 3NB UK; Red de Grupos de Investigación Recursos Faunísticos, Facultad de Veterinaria, Universidad de Extremadura, 10003 Cáceres, Spain; Spanish National Center of Biotechnology (CSIC), C/Darwin 3, Campus de Cantoblanco, 28049 Madrid Spain; Department of Virology, Animal and Plant Health Agency, Addlestone, Surrey, KT15 3NB UK; Department of Pathology, Animal and Plant Health Agency, Addlestone, KT15 3NB UK; Department of Pathology and Infectious Diseases, School of Veterinary Medicine, University of Surrey, Guildford, GU2 7TE UK

## Abstract

Porcine reproductive and respiratory syndrome virus (PRRSV) induces a weak immune response enabling it to persist in different organs of infected pigs. This has been attributed to the ability of PRRSV to influence the induction of cytokine responses. In this study, we investigated the cytokine transcriptional profiles in different compartments of the mediastinal lymph node of pigs infected with three genotype 1 PRRSV strains of differing pathogenicity: the low virulence prototype Lelystad virus (LV), and UK field strain 215–06 and the highly virulent subtype 3 SU1-Bel isolate from Belarus. We have used a combination of laser capture micro-dissection (LCM) followed by real time quantitative PCR (RT-qPCR) and immunohistochemical (IHC) detection of immune cell markers (CD3, CD79a and MAC387) and RT-qPCR quantification of PRRSV and cytokine transcripts. Compared to mock infected pigs, we found a significant downregulation of TNF-α and IFN-α in follicular and interfollicular areas of the mediastinal lymph node from 3 days post-infection (dpi) in animals infected with all three strains. This was accompanied by a transient B cell depletion and T cell and macrophage infiltration in the follicles together with T cell depletion in the interfollicular areas. A delayed upregulation of IFN-γ and IL-23p19 was observed mainly in the follicles. The PRRSV load was higher in all areas and time-points studied in the animals infected with the SU1-Bel strain. This paper describes the first application of LCM to study the cytokine transcript profiles and virus distribution in different compartments of the lymph node of pigs.

## Introduction

Porcine reproductive and respiratory syndrome (PRRS) is characterized by respiratory disease in neonatal and growing pigs and reproductive failure in gilts and sows (increased number of abortions, mummified foetuses, stillbirth and weak-born piglets) [1-3]. PRRS is considered one of the most economically important swine infectious diseases around the world, with estimated losses of up to $664 million annually in the USA alone [4,5].

PRRS virus (PRRSV) is classified into two genotypes, type 1 (European or PRRSV-1) and type 2 (North American or PRRSV-2) [6]. In addition, a high degree of genetic variation in both genotypes has been found, with PRRSV-1 having been divided into 3 subtypes: pan-European subtype 1 and East European subtypes 2 and 3 [7], with the possibility of a fourth subtype being suggested [8]. Significant differences in virulence have also been described between PRRSV-1 subtypes, with the East European subtype 3 seemingly comprising the most virulent strains [9-11].

PRRSV shows a marked tropism for cells of the monocyte-macrophage lineage [12]. The main target of PRRSV are alveolar and other tissue macrophages, and to a lesser extent, monocytes and dendritic cells [13]. Absent or weak interferon alpha (IFN-α) secretion [14] and a consequent weak and delayed cell-mediated immune response with low levels of IFN-γ has been described following PRRSV infection [15,16]. Pigs infected with PRRSV have also shown a delayed production of neutralizing antibodies [17]. PRRSV replication has been reported in lymphoid organs [18,19] however studies have also shown a lack of homogeneity in proinflammatory cytokine responses [20-22]. This suggests a role for this tissue in the pathogenesis of PRRS but also highlights the need for comparative in vivo studies using PRRSV-1 strains which differ in their virulence. The porcine lymph node (LN) has a dense medulla, where T cells are predominant and which lacks sinuses and cords. The LN cortex is divided into two differentiated areas the follicles (F) and interfollicular (IF) areas. The F is a B cell rich area that also contains follicular dendritic cells (fDC) and CD4^+^ T helper cells that collaborate in antigen presentation to B cells and subsequent antibody production. The IF area is rich in T cells [21,22]. It has been proposed that the immunopathogenesis of porcine circovirus 2 (PCV2) infection is associated with follicular changes in lymph nodes [23], and it is suggested that this could also be the case for PRRSV infection.

Transcriptional expression profiling studies can aid to the understanding of infection biology and the molecular basis of disease [24]. Several studies have analysed the host transcriptional profiles during PRRSV infection in different organs by taking small pieces of tissue [25-28], but none have addressed transcriptional profiling in defined tissue structures. Laser capture microdissection (LCM) is a powerful tool for the acquisition of homogeneous cell populations or specific tissue structures which can be analyzed by a variety of molecular biology techniques and aid disease pathogenesis investigations [29-31].

The main aim of this study was to compare the cytokine transcriptional profiles in different compartments of lymph nodes from pigs infected with three PRRSV-1 strains of defined virulence. This study included the prototype Lelystad virus (LV) and a field strain from the UK (215–06), both categorized as low virulence subtype 1 viruses, and a divergent and highly pathogenic Eastern European subtype 3 strain from Belarus (SU1-Bel). Using LCM followed by quantitative reverse transcriptase PCR (RT-qPCR), viral RNA and cytokine transcripts were measured in the F and IF areas of mediastinal lymph nodes (Med-LN) collected at selected time-points post-infection. To further contextualize these data, T cells, B cells and macrophages were immunolabelled to characterize these cell populations within the lymph node compartments.

## Material and methods

### Viruses

Three PRRSV-1 strains were used in this study: the LV-Ter Huurne was selected as the prototype subtype 1 strain (kindly provide by Anne Marie Rebel, CVI Lelystad, The Netherlands) [11]; the subtype 1 215–06 strain was isolated in 2006 from the serum of a post-weaning piglet showing signs of wasting and poor condition on a farm in England and isolated at the Animal and Plant Health Agency (APHA, Addlestone, UK); and the highly pathogenic subtype 3 strain SU1-Bel, isolated at the APHA from lung tissue of a pig from Belarus (kindly provided by Dr Tomasz Stadejek, Warsaw University of Life Sciences, Poland) was also included. Virus propagation was carried out as previously described [10].

### Animals and experiment design

Seventy-six specific-pathogen-free five-week-old male piglets from a PRRSV and PCV2 seronegative farm in the Netherlands were used in this study. These animals were matched by weight and randomly allocated to four groups; for the control group 16 animals were allocated, whereas for each infected group 20 animals were used. Each group was housed in separate rooms, which allowed the free airflow from the outside, and were allowed to acclimatise for 14 days prior to the experiment. Measures were taken to prevent cross-contamination between groups, including the change of clothes and equipment between each room. At seven weeks of age, 3 groups of piglets were inoculated intranasally with 1.5 mL of complete Roswell Park Memorial Institute medium supplemented with 10% FBS (cRPMI) containing 10^5^ 50% tissue-culture infective dose (TCID_50_) of PRRSV-1 strain (LV, 215–06 and SU1-Bel, respectively). The remaining control group was inoculated intranasally with 1.5 mL of uninfected porcine alveolar macrophage cryolysate in cRPMI. Four animals in the control group and five from each virus-inoculated group were euthanized at 3 and at 7 days post-infection (dpi). All remaining animals were euthanized at 35 dpi. This experiment was performed in accordance with the Animals (Scientific Procedures) Act, 1986, UK, following approval of the APHA Ethical Review Committee. A clinical scoring system with predefined humane endpoints was used to prevent undue suffering.

### Clinical signs, gross pathology and histopathology

Pigs were monitored daily throughout the study, and clinical signs, including rectal temperatures, were scored and recorded as previously described [10]. The Med-LN is the main draining lymph node for the apical and medial lobes of the lungs. Since PRRSV is most frequently detected in these lung lobes [32], Med-LN was selected for this study and the gross pathology evaluated during post-mortem examination. For the analysis by RT-qPCR, a piece of 10×10×3 mm of Med-LN was embedded and cryopreserved in optimal cutting temperature (OCT) compound (Sakura Finetek Europe B.V., The Nederland) as previously described [33]. For histopathology examination, the remaining Med-LN samples were fixed in 10% buffered formalin, routinely processed, embedded in paraffin-wax, and 4 μm tissue sections stained with haematoxylin and eosin. The histopathological lesions were evaluated in these slides using a light microscope.

### Immunohistochemistry (IHC)

The immunolabelling of cell markers was done using the avidin-biotin complex method (ABC Vector Elite, Vector laboratories, USA) as described previously [19,34]. Briefly, 4 μm thick sections were dewaxed and rehydrated, followed by endogenous peroxidase inhibition with 3% H_2_O_2_ in methanol for 30 min. Depending on the epitope of interest, antigen retrieval in the tissue sections was performed by enzymatic trypsin/alpha-chymotrypsin (for CD3 and MAC-387) or by microwaving the sections in citric acid pH6.0 (for CD79a) or pH6.0 citrate buffer (for PRRSV nucleocapsid N protein). The slides were mounted in a Sequenza Immunostaining Centre (Shandon Scientific, UK) and washed with Tris buffered saline (TBS; pH 7.6, 0.005 M; Sigma–Aldrich, UK) and incubated for 30 min at room temperature with 100 μL per slide of blocking solution. The primary antibodies used were monoclonal anti-human CD3 (1:1000; Dako, UK), monoclonal anti-human CD79a (1:400; Dako, UK), and monoclonal anti-human MAC-387 (1:100; AbDSerotec, UK). Each antibody was applied for 1 h at room temperature. In each case, the corresponding biotinylated secondary antibody (Vector Laboratories, UK) was then incubated for 30 min at room temperature. Slides were then incubated for 30 min with avidin-biotin complex and labelling performed using 3,30-diaminobenzidine tetrahydrochloride (DAB; Sigma-Aldrich, UK). Sections were counterstained with Mayer’s haematoxylin, dehydrated and mounted. Positive and negative controls, as well as isotype controls, were included in each IHC run.

The immunolabelled Med-LN sections were examined by light microscopy, and immunolabelling measurements recorded using a score ranking from −3 to 0 (cellular depletion) and from 0 to 3 (cellular increment) compared to the control group. Positive scale: 0 = absence (<1 positive cell/structure); 1 = scarce (1–10 positive cells/structure); 2 mild-moderate (11–30 positive cells/structure); 3 abundant (>31 positive cells/structure). Negative scale: 0 = absence (no different to control group); −1 = scarce (5% less positive cells compares with control group); −2 mild-moderate (6-10% less positive cells compares with control group); −3 abundant (more than 10% less positive cells compares with control group).

### Laser capture microdissection (LCM)

Frozen OCT-embedded Med-LNswere cut to 10 μm thick sections using a cryostat. Tissue sections were placed on membrane-coated slides (PEN-Membrane 2.0 μm; Leica Microsystems, Germany). The cryostat was treated with RNAZap Solution (Life Technologies, UK) between each sample in order to avoid cross-contamination of RNA. Sections were air-dried and fixed in 70% ethanol for 5 min and stained with RNase free haematoxylin for 1 min. Two consecutive Med-LN tissue sections were used per animal. All observed F and peripheral IF were dissected and captured separately in RNase-free PCR tubes (Greiner bio-one, UK) using a laser microdissector (Leica LMD6500, Leica, Germany). The samples were immediately frozen and stored at −80 °C until laboratory processing.

### RNA extraction and reverse transcription quantitative polymerase chain reaction (RT-qPCR)

For each sample, total RNA was extracted using the RNA queous-Micro Kit (Ambion,Life Technologies, UK) following the manufacturer’s instructions for LCM samples, including the DNase I treatment and DNase inactivation steps. The total RNA was quantified using Qubit 2.0 Fluorometer (Life Technologies) and all samples adjusted to an RNA concentration of 2 ng/μL).

PRRSV infection kinetics in the F and IF areas of Med-LN were studied by measuring viral RNA. PRRSV RT-qPCR was performed as previously described [35]. Briefly, 2 μL of sample or standard PRRSV-1 RNA dilutions were added as template to the QuantiTec Probe RT-PCR Kit (Qiagen, UK) following the manufacturer’s instructions for a total volume of 25 μL. Data was analysed by changes in the cycle threshold (Ct), and results were calculated as 38 – Ct, which represented the difference between the last cycle of the PRRSV RT-qPCR and the Ct for each sample.

Primers and TaqMan probe sets for porcine cytokines were synthesized by Sigma-Aldrich (Table 1). All cytokines primer pairs produced amplicons smaller than 150 base pairs (bp). All primer and probe sets were optimized for our laboratory conditions.

**Table 1:** **Primers and probes used for qPCR**

**Gene**	**Primer Forward**	**Primer Reverse**	**Probe**
	**nM**	**nM**	**nM**
**β-Actin**	5´-cactcctaacgctgtggatcag-3´	5´-ccacttaactatcttgggcttatcg-3´	5´-[6FAM]-cacgtgcttcacgcggcagc-[TAM]-3´
	300	300	50
**TNF-α**	5´-tggccccttgagcatca-3´	5´-cgggcttatctgaggtttgaga-3´	5´-[6FAM]-ccctctggcccaaggactcagatca-[TAM]-3´
	900	600	50
**IFN-α**	5´-tcagctgcaatgccatctg-3´	5´-agggagagattctcctcatttgtg-3´	5´-[6FAM]-tgacctgcctcagacccacagcc-[TAM]-3´
	150	600	50
**IFN-γ**	5´-gaaaagctgattaaattccggtag-3´	5´-aggttagatcttggtgacagatc-3´	5´-[6FAM]-tctgcagatccagcgcaaagccatcag-[TAM]-3´
	300	900	50
**SOCS1**	5´-ttcttcgccctcagtgtgaa-3´	5´-ggcctggaagtgcacgc-3´	5´-[6FAM]-ttcgggccccacaagcatcc-[TAM]-3´
	300	300	50
**IL-10**	5´-tgagaacagctgcatccacttc-3´	5´-tctggtccttcgtttgaaagaaa-3´	5´-[6FAM]-caaccagcctgccccacatgc-[TAM]-3´
	300	300	150
**TGF-β**	5´-agggctaccatgccaattt-3´	5´-ccgggttgtgctggttgt-3´	5´-[6FAM]-cactcagtacagcaaggtcctggctctgta-[TAM]-3´
	600	600	50
**IL-23p19**	5´-agaagagggagatgatgagac-3´	5´-agcaggactgactgccgtcc-3´	5´-[6FAM]-ctgaggatcacagccatccccgc-[TAM]-3´
	900	300	50

In order to obtain complementary DNA (cDNA) reverse transcription was carried out using the SuperScript VILO^TM^ cDNA Synthesis Kit (Applied Biosystems, UK) and cDNA was stored at −80 °C until laboratory processing. 2 μL of cDNA diluted 1:100 in RNase free water was used as template for each cytokine qPCR, using the EXPRESS qPCR Supermix (Invitrogen) with ROX reference dye in a total volume of 20 μL. Thermal cycling conditions were 2 min at 95 °C, 45 cycles of denaturation at 95 °C for 15 s and annealing/extension at 60 °C for 1 min. Reverse transcription negative controls and non-template controls were included. PCR plates were centrifuged prior to amplification using the Strategene MX3000P qPCR System (Stratagene, UK). Fold-change of cytokine expression was calculated using the 2^-ΔΔCt^ method [36] and β-actin was used as the endogenous calibrator; which was selected due to its stable expression in porcine lymphatic tissue [37]. Relative gene expression results are presented on a base 2 logarithmic scale.

### Statistical analysis

Statistical analyses were performed using SPSS 15.0 software (SPSS Inc., USA) and graphs were prepared using SPSS 15.0 software (SPSS Inc., USA) and GraphPad Prism 5 (GraphPad Software, Inc., USA). To determine the non-normal distribution of all data a Kolmogorov-Smirnov test was used. Non-normal distributed data was then analysed using a non-parametric test. The Kruskal-Wallis test was performed to analyse the mean parameters (PRRSV RNA load, immunolabelled cells and cytokine expression) between the animal groups (Control, LV, 2015–06, and SU1-Bel) at three different time-points post inoculation (3 dpi, 7 dpi, and 35 dpi). Thereafter, we performed multiple comparisons using the non-parametric Mann–Whitney-U test with Bonferroni correction as post-hoc to determine significant individual differences. Finally, the presence of differences between results obtained in F and IF tissues were assessed using the non-parametric Wilcoxon signed-rank test. The correlations between PRRSV RNA and cytokine transcripts were determined by Spearman’s Rho analysis. A *p* value of less than 0.05 was considered significant.

## Results

### Clinical signs, gross pathology and histopathology

Rectal temperatures remained within the physiological range in control as well as infected animals, except for SU1-Bel infected animals that showed hyperthermia at 3, 8–10 dpi (over 40 °C). Only SU1-Bel infected animals developed elevated clinical sign scores (mean score from 5 to 10) compared with the rest of animals in this study (mean clinical score only up to 3) [10]. Macroscopically at post-mortem, a mild to moderate enlargement of Med-LN was observed in infected animals at 3 and 7 dpi, while at 35 dpi no Med-LN damage was observed [10]. The control animals did not show any lesions during the study. The histopathological analysis of Med-LN revealed the presence in the lymphoid follicles of a mild hypertrophy of germinal centres and the presence of apoptotic bodies at 3 and 7 dpi. At 35 dpi virus-infected groups showed no significant histological lesions.

### PRRSV RNA quantification

**Figure 1:**
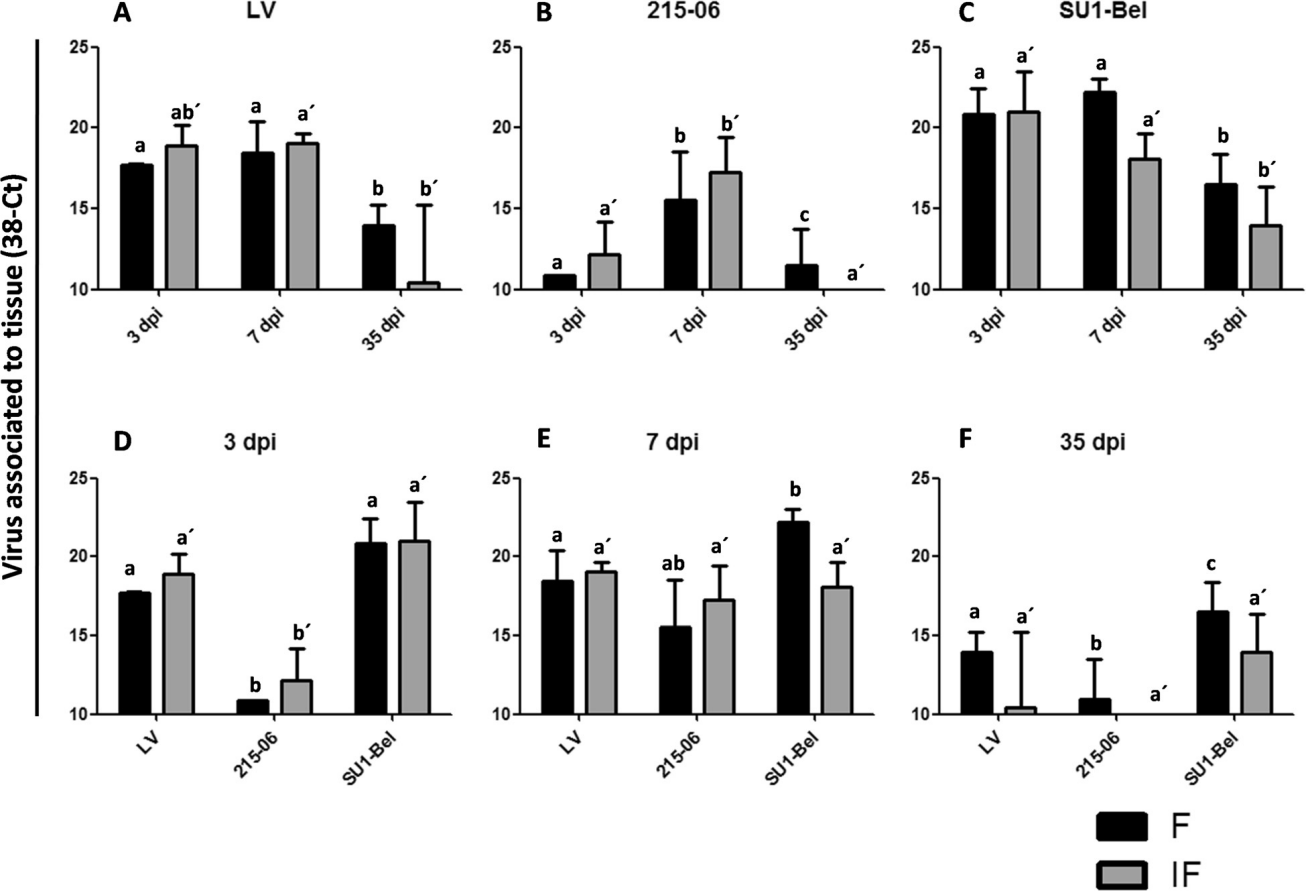
**PRRSV viral load in mediastinal lymph node.** PRRSV RNA was quantified by RT-qPCR and data represented by changes in the cycle threshold (Ct) in F and IF areas of Med-LN of LV **(A)**, 215–06 **(B)** and SU1-Bel **(C)** infected animals. Differences between distinct PRRSV-1 infected groups are showed for 3 **(D)**, 7 **(E)**, and 35 **(F)** days post-infection. This figure showed the median of each group ± SD. In control animals the viral RNA were not detected (data not shown). Identical superscript letters indicate no significant difference (*p* > 0.05), whereas different superscript letters indicate statistically significant differences (*p* < 0.05) between the same compartment among different groups (a, b, and c for follicle; a´, b´, and c´ for interfollicular area).

All PRRSV RNA measurements are shown in Figure 1. All PRRSV infected animals showed a higher PRRSV RNA load at 3 and 7 dpi than at 35 dpi (*p* < 0.05). No viral RNA was detected in control animals (data not shown). The LV group showed at 3 dpi higher viral RNA in the IF area of Med-LN (38 - Ct = 18.89 ± 1.27 SD; *p* < 0.05). At 7 dpi the viral RNA amount was similar between Med-LN compartments (38-Ct = 18.89 ± 1.90 SD; 38 - Ct = 19.09 ± 0.58 SD; F and IF areas respectively). At 35 dpi the LV RNA was significantly reduced in Med-LN compartments (38 - Ct = 13.98 ± 1.23; 38 - Ct = 10.46 ± 4.76; F and IF area respectively; *p* < 0.05) (Figure 1A).

The PRRSV-1 215–06 RNA was detected at 3 dpi mainly in IF area of Med-LN (38 - Ct = 12.16 ± 2.02; *p* < 0.05). At 7 dpi the 215–06 RNA increased to the highest level seen in the study in this group, and was higher in IF area than in F (38 - Ct = 17.27 ± 2.16; 38 – Ct = 15.55 ± 2.96; respectively; *p* < 0.05). Interestingly the 215–06 virus was only detected in F of Med-LN at 35 dpi (38 - Ct = 10.94 ± 2.55; *p* < 0.05) (Figure 1B).

At 3 dpi PRRSV-1 SU1-Bel RNA was mostly detected in IF (38 - Ct = 21.03 ± 2.43; *p* < 0.05), whereas at 7 dpi SU1-Bel RNA was mostly found in F (38 - Ct = 22.20 ± 0.85; *p* < 0.05). At 35 dpi the SU1-Bel RNA decreased but was statistically significant in both Med-LN compartments although predominantly detected in F of Med-LN (38 - Ct = 16.49 ± 1.88; *p* < 0.05) (Figure 1C).

The SU1-Bel group showed the higher load of viral RNA in both Med-LN compartments at each time-point throughout this study (Figures 1D-F; *p* < 0.05). At 3 dpi, PRRSV RNA was mainly detected in IF areas (Figure 1D), where the 215–06 RNA amount was statistically significant lower than in LV and SU1-Bel groups (*p* < 0.05). PRRSV RNA remained higher in IF compartment at 7 dpi for LV and 215–06 groups, but it was higher in F of SU1-Bel-infected pigs (*p* < 0.05; Figure 1E). By 35 dpi, the SU1-Bel RNA in F was statistically significant higher than in LV infected group (*p* < 0.05), and in both groups it was significant higher than in F of Med-LN of 215–06 infected animals (*p* < 0.05; Figure 1F).

### Immunohistochemistry (IHC)

**Figure 2:**
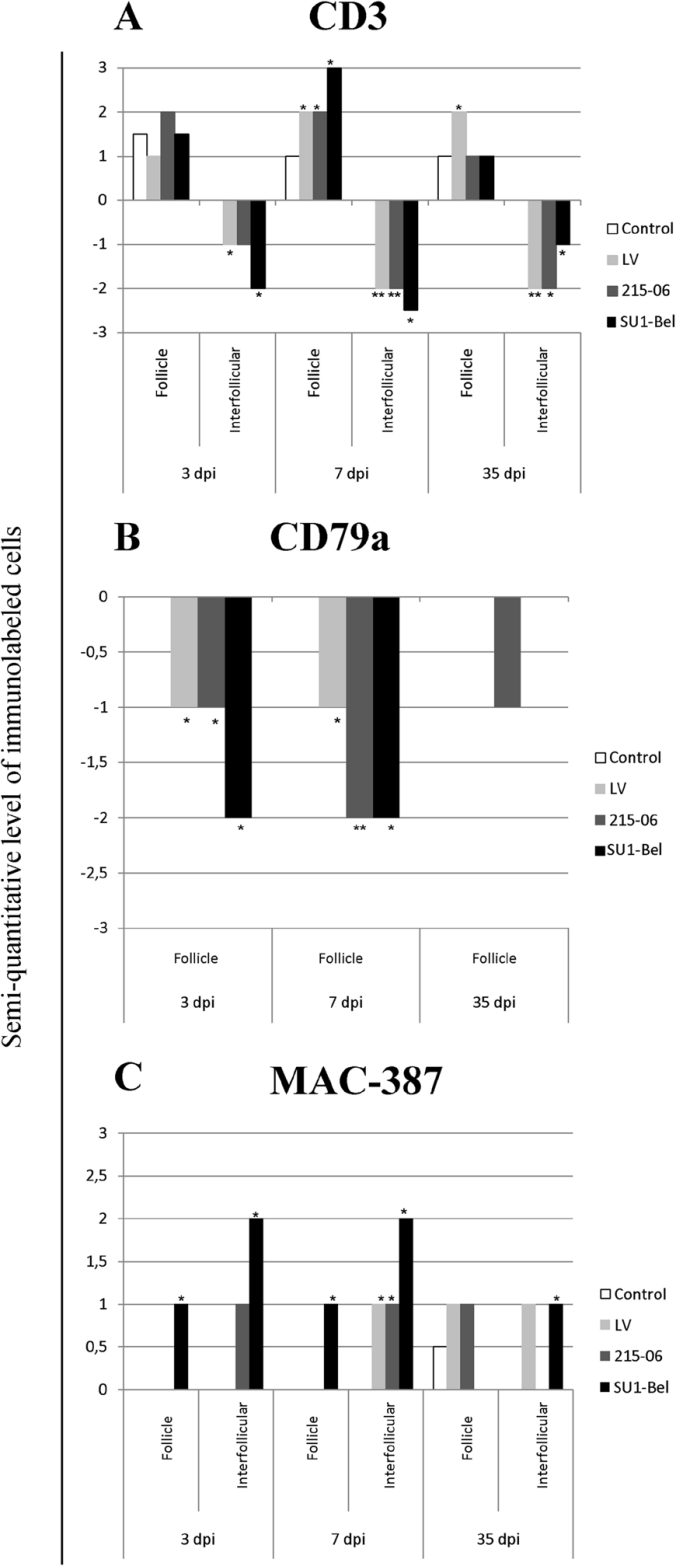
**Immunolabelled cell populations in mediastinal lymph node of PRRSV-1 infected pigs. A**. CD3 staining for T cells detection; it was observed a general T cell depletion during this study for all PRRSV infected groups, otherwise the T cell immunolabeled cells increased in F at 7 dpi. **B**. CD79a staining for B cell staining in follicle, where was evidence a B cell depletion at 3 and 7 dpi for all PRRSV infected animals. **C**. Macrophage detection with MAC-387 staining, the amount of immunolabeled macrophages increased along this study in SU1-Bel infected pigs. The bars represent s-qic mean values; asterisks indicated the statistically significant differences (*p* < 0.05) against the control group. It is important to note that only a few animals in the control groups showed a higher number of immunolabeled cells in the follicle compared to the rest of animals within this group. For this reason there is a bar in some control groups.

CD3 immunolabelling defined the T cell population locations in Med-LN [38,39]. During this study the number of T cells was found to be increased in F of Med-LN at 7 dpi in all infected animals compared with the control animals (2 semi-quantitative immunolabelled cells [s-qic] for LV and 215–06 groups and 3 s-qic for the SU1-Bel group; *p* < 0.05). At 35 dpi this follicular T cell population remained statistically higher in LV infected animals (2 s-qic for LV; *p* < 0.05; Figure 2A). Conversely, the number of T cells decreased in the Med-LN IF from 3 dpi in LV (−1 s-qic; *p* < 0.05) and SU1-Bel inoculated animals (−2 s-qic; *p* < 0.05), remaining lower than control animals at 7 and 35 dpi for all infected animals (−2 s-qic for LV and 215–06, and from −2.5 to −1 s-qic for SU1-Bel; *p* < 0.05; Figure 2A).

The immunolabelling of the pan-B cell surface protein CD79a [40,41] showed B cells only in the F of Med-LN. In all infected animals these cells decreased in number compared with control animals at 3 dpi (−1 s-qic for LV and 215–06 and −2 s-qic for SU1-Bel groups; *p* < 0.05) and 7 dpi (−1 s-qic for LVand −2 s-qic for both 215–06 and SU1-Bel groups; *p* < 0.05). At 35 dpi the B cell population did not show a statistically significant change between the infected and control groups (Figure 2B).

Macrophages were identified through MAC-387 imunolabelling of Med-LN sections [21,42]. In the F, macrophage numbers were found to be statistically increased in SU1-Bel infected animals at 3 and 7 dpi (1 s-qic on both days; *p* < 0.05). In the IF areas of SU1-Bel infected animals, the macrophage population was increased at all time-points (from 2 to 1 s-qic; *p* < 0.05). However the animals inoculated with LV and 215–06 PRRSV strains showed a smaller increase in macrophages in the IF area only at 7 dpi (1 s-qic; *p* < 0.05; Figure 2C).

**Figure 3:**
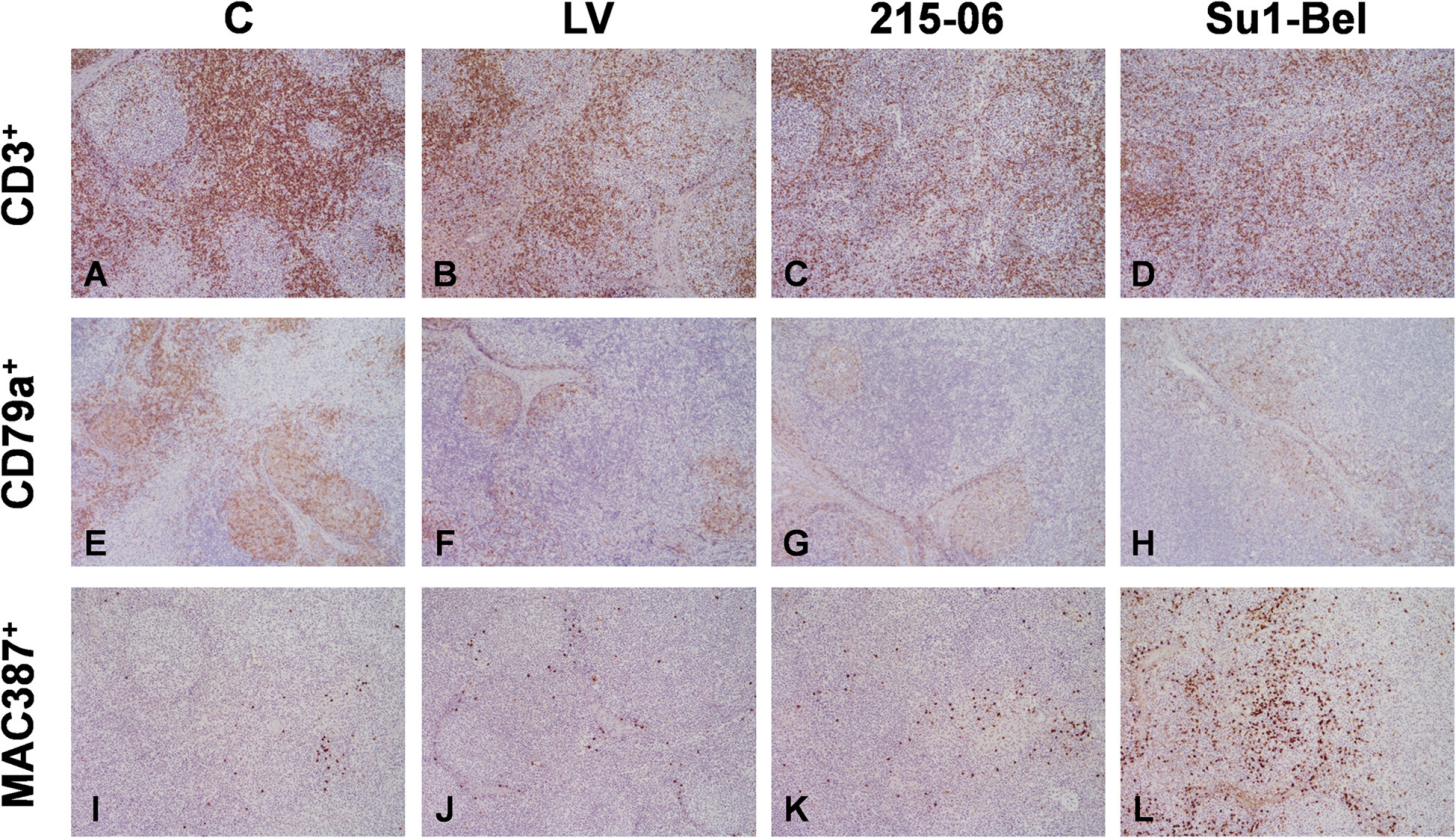
**Representative images of CD3, CD79a and MAC387 IHC staining in mediastinal lymph node.** CD3 **(A, B, C, D)**, CD79a **(E, F, G, H)** and MAC387 **(I, J, K, L)** in mediastinal lymph nodes of control pigs **(A, E, I)** and infected with LV **(B, F, J)**, 215–06 **(C, G, K)** and SU1-Bel **(D, H, J)** strains, at 7 dpi. An increased in the number of CD3^+^ cells is observed in the lymphoid follicles from all infected groups together with a depletion of these cells in the interfollicular areas. A decrease in the number of CD79a cells in the follicles is also observed in all the infected groups. A substantial increase in the number of MAC387 is observed in the follicles and interfollicular areas from SU1-Bel infected animals **(L)** together with a mild increase in the LV **(J)** and 215–06 **(K)** groups. Original magnification: 20x.

Representative images of CD3, CD79a and MAC387 IHC staining in all the groups are shown in Figure 3.

### Cytokine gene expression in Med-LN compartments

**Figure 4:**
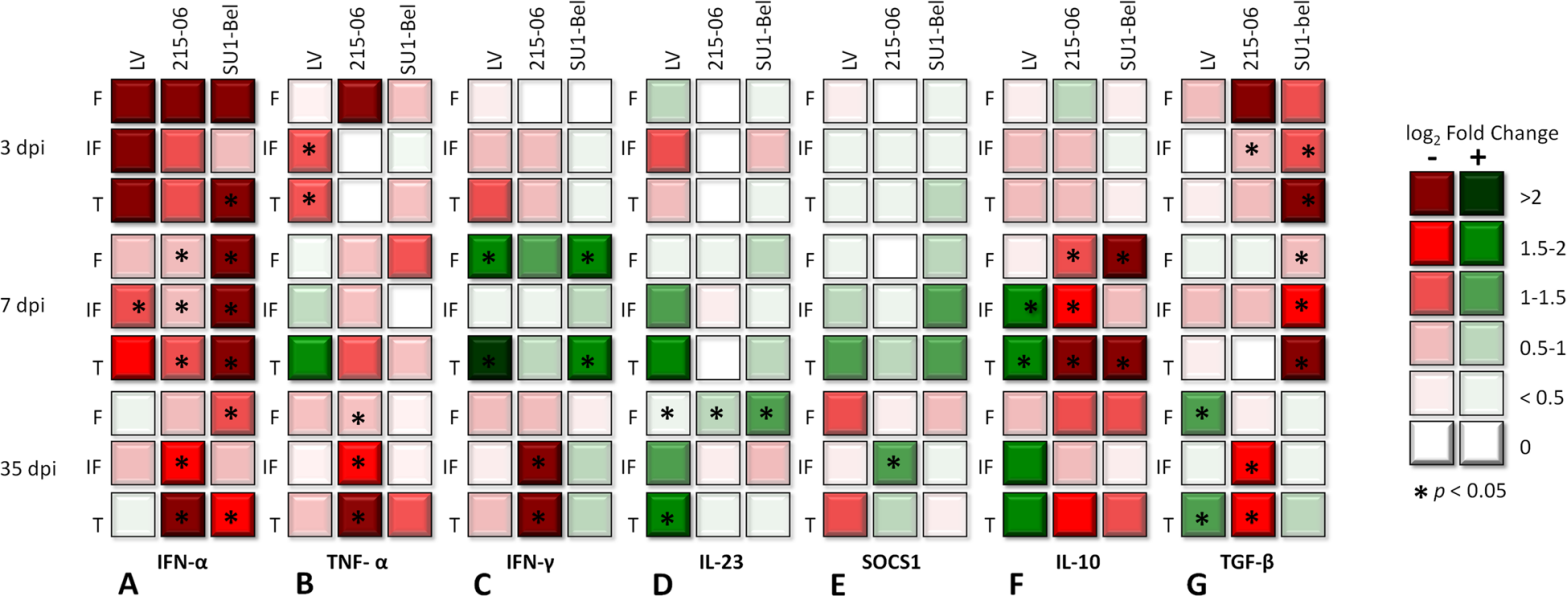
**Cytokine gene expression in mediastinal lymph node compartments.** Log 2 Fold change in transcript level of IFN-α **(A)**, TNF-α **(B)**, IFN-γ **(C)**, IL-23p19 **(D)**, SOCS1 **(E)**, IL-10 **(F)** and TGF-β **(G)** gene expression relative to the control gene β-Actin; which was calculated by 2^-ΔΔCt^ method. Log_2_ fold change of gene expression in follicle **(F)**, interfollicular area (IF) or total Med-LN lymph node (T) values are depicted. Green boxes indicate upregulation and red boxes indicate downregulation as the key showed. The statistically significant differences (*p* < 0.05) with control group are indicated by asterisks on the boxes.

To assess immune responses against the selected PRRSV-1 strains within the different Med-LN compartments, IFN-α, TNF-α, IFN-γ, IL-23p19, SOCS1, IL-10 and TGF-β transcript levels were measured by RT-qPCR and compared against control animals (Figure 4). The suitability of the RNA quality was confirmed by testing expression of β-actin in the samples. All cDNA samples produced a positive amplification to each gene by qPCR.

Compared to control animals, in both studied LN compartments the IFN-α was generally downregulated in all infected groups and time-points, which was statistically significant for all PRRSV-1 strains at 7 dpi (*p* < 0.05), when the SU1-Bel group showed the lowest IFN-α transcript levels. The IFN-α remained downregulated at 35 dpi in the Med-LN of 215–06 and Su1-Bel infected pigs (*p* < 0.05; Figure 4A). TNF-α expression was downregulated in IF area of Med-LN of LV infected pigs at 3 dpi (*p* < 0.05) and in Med-LN of 215–06 group at 35 dpi (*p* < 0.05; Figure 4B) compared with the control groups. IFN-γ was significantly upregulated in F of Med-LN of LV (*p* < 0.05) and SU1-Bel infected pigs at 7dpi (*p* < 0.05); on the other hand the IFN-γ was significant downregulated in IF area of Med-LN of 215–06 infected animals at 35 dpi (*p* < 0.05; Figure 4C). At 35 dpi, IL-23p19 was statistically significant upregulated in F of all infected groups (*p* < 0.05; Figure 4D). No significant differences in the expression IL-12 mRNA were detected during the study (data not shown). SOCS1 transcript levels were not statistically significantly regulated for any infected group during the first week of this experiment. However at 35 dpi, SOCS1 transcript levels were increased in the IF area of the Med-LN of animals from the 215–06 group (*p* < 0.05; Figure 4E). At 7 dpi the IL-10 gene expression was significantly downregulated in the F of both 215–06 and SU1-Bel groups (*p* < 0.05) and in the IF area (*p* < 0.05; only 215–06 group). In contrast, IL-10 was significantly upregulated in IF area of Med-LN of the LV infected group (*P* < 0.05; Figure 4F). TGF-β was statistically significantly downregulated at 3 dpi in IF areas of Med-LN of 215–06 and SU1-Bel infected animals (*p* < 0.05), at 7 dpi in Med-LN of SU1-Bel group (*p* < 0.05), and at 35 dpi in IF area of Med-LN of 215–06 infected pigs (*p* < 0.05); contrary, it was statistically upregulated in F of the LV group at 35 dpi (*p* < 0.05; Figure 4G).

The statistically significant correlations between gene expression of immune genes in Med-LN compartments and LV, 215–06 and SU1-Bel PRRSV RNA are respectively represented in the Tables 2, 3 and 4.

**Table 2:** **Correlations between LV RNA and the gene expression of TNF-α, IFN-α, IFN-γ, SOCS1, IL-23p19, IL-10 and TGF-β**

		**IFN-α**	**IFN-γ**	**IL-23**	**SOCS1**	**IL-10**	**TGF-β**	**ORF7 PRRSV**
**TNF-α**	F	0.857**	0.094	−0.102	0.064	0.160	0.964**	−0.077
	IF	0.184	0.469	0.643*	0.471	0.687**	0.479	−0.190
	T	0.750**	0.248	0.031	0.091	0.305	0.921**	−0.043
**IFN-α**	F		−0.255	−0.351	0.009	−0.201	0.901**	−0.110
	IF		0.307	0.276	−0.086	0.094	0.619*	−0.663**
	T		−0.142	−0.156	−0.132	0.167	0.880**	−0.301
**IFN-γ**	F			0.342	0.607*	0.131	−0.082	0.413
	IF			0.220	0.680**	0.100	0.282	0.144
	T			0.167	0.649**	0.222	0.008	0.438
**IL-23**	F				0.239	0.112	−0.179	−0.208
	IF				0.109	0.553*	0.183	−0.231
	T				−0.004	0.395	−0.106	−0.154
**SOCS1**	F					0.809	−0.063	0.527*
	IF					0.310	−0.016	0.303
	T					0.211	−0.139	0.486
**IL-10**	F						−0308	0.397
	IF						0.116	−0.332
	T						0.144	−0.151
**TGF-β**	F							−0.206
	IF							−0.557*
	T							−0.245

*p < 0.05; **p < 0.01. F (follicle of Med-LN), IF (interfollicular area of Med-LN) and T (Total cortical compartments of Med-LN).

**Table 3:** **Correlations between 215–06 RNA and the gene expression of TNF-α, IFN-α, IFN-γ, SOCS1, IL-23p19, IL-10 and TGF-β**

		**IFN-α**	**IFN-γ**	**IL-23**	**SOCS1**	**IL-10**	**TGF-β**	**ORF7 PRRSV**
**TNF-α**	F	0.66	−0.015	0.679*	0.462	−0.169	0.919**	0.099
	IF	−0.149	0.490*	0.239	0.081	0.204	0.438	0.133
	T	−0.010	0.349	0.380	0.123	0.254	0.386	−0.024
**IFN-α**	F		−0.316	−0.482	−0.445	−0.666*	0.073	0.129
	IF		0.145	0.091	−0.399	−0.137	0.188	0.252
	T		−0.077	0.070	−0371	−0.476*	0.378	0.205
**IFN-γ**	F			0.016	0.193	0.257	0.067	0.612*
	IF			0.002	−0.385	−0.123	0.808**	0.661**
	T			−0.068	0.133	0.178	0.436	0.524*
**IL-23**	F				0.233	0.266	0.526	−0.208
	IF				0.135	0.324	−0.225	0.017
	T				0.395	0.403	0.070	−0.022
**SOCS1**	F					0.362	0.420	−0.013
	IF					0.374	−0.267	−0.221
	T					0.560*	0.206	−0.201
**IL-10**	F						−0.173	−0.114
	IF						−0.261	−0.334
	T						0.124	−0.341
**TGF-β**	F							0.235
	IF							0.582*
	T							0.276

*p < 0.05; **p < 0.01. F (follicle of Med-LN), IF (interfollicular area of Med-LN) and T (Total cortical compartments of Med-LN).

**Table 4:** **Correlations between SU1-Bel RNA and the gene expression of TNF-α, IFN-α, IFN-γ, SOCS1, IL-23p19, IL-10 and TGF-β**

		**IFN-α**	**IFN-γ**	**IL-23**	**SOCS1**	**IL-10**	**TGF-β**	**ORF7 PRRSV**
**TNF-α**	F	−0.047	−0.146	0.651**	−0.254	0.292	0.471	−0.683**
	IF	−0.419	−0.188	0.107	−0.211	−0.404	0.167	−0.240
	T	−0.138	−0.165	0.436	−0.164	−0.181	0.266	−0.549*
**IFN-α**	F		−0.401	0.060	−0.449	0.165	0.649**	−.273
	IF		−0.186	−0.557*	−0.258	0.339	0.193	−0.044
	T		−0.402	−0.250	−0.629*	0.373	0.433	−0.265
**IFN-γ**	F			−0.040	0.432	−0.327	−0.229	0.353
	IF			0.298	0.300	−0.347	−0.079	0.082
	T			0.147	0.476	−0.169	−0.267	−0.74
**IL-23**	F				−0.037	0.004	0.455	−0.416
	IF				0.594*	−0.318	−0.216	−0.124
	T				0.496*	−0.178	−0.573*	−0.530*
**SOCS1**	F					−0.406	−0.481	0.515*
	IF					0.002	−0.341	−0.245
	T					−0.121	−0.181	−0.089
**IL-10**	F						0.088	−0.384
	IF						−0.283	−0.108
	T						−0.246	−0.093
**TGF-β**	F							−0.541*
	IF							0.052
	T							−0.263

*p < 0.05; **p < 0.01. F (follicle of Med-LN), IF (interfollicular area of Med-LN) and T (Total cortical compartments of Med-LN).

## Discussion

This study reveals additional data generated from an animal experiment conducted at APHA as part of the EU FP7 PoRRSCon project [10,43]. This study was conceived to directly compare the modulation of immune response transcripts from defined Med-LN compartments of pigs experimentally infected with PRRSV-1 strains of varying virulence.

Pigs infected with the SU1-Bel PRRSV strain have shown greater Med-LN gross pathology compared with the pan-European subtype 1 strains LV and 215–06 [10]. In the present study the highest PRRSV RNA levels were detected in the IF area of Med-LN, which suggest that PRRSV was entering from the apical and medial lung lobes [32] to the Med-LN via draining lymphatics vessels. We have previously shown that for all PRRSV infected groups, the virus load in serum showed a peak at 7 dpi decreasing until 28 dpi, whereas in bronchoalveolar lavage fluid (BALF) the virus persisted until 35 dpi [10]. Interestingly, despite its enhanced virulence the levels of virus in both circulation and BALF were lowest in SU1-Bel infected animals. In contrast, in the present analysis we have detected the higher levels for SU1-Bel in Med-LN compared with the subtype 1 strains, which suggest that this PRRSV-1 subtype 3 strainmay infect LN resident/transient cell populations more efficiently and this could be linked to the higher pathogenicity of this strain. In support of this data, the PRRSV N protein has been detected by IHC mainly in the cytoplasm of macrophages of the Med-LN of infected pigs from on 3, 7 and 35 dpi, with highest levels of immunolabelled cells found in SU1-Bel infected animals [43].

The use of LMD in the present study, enabled for the first time the measurement of PRRSV-1 RNA within different Med-LN compartments. The results showed the presence of PRRSV in the F of the Med-LN at 35 dpi albeit at low levels, according with a previous report that described the detection of infectious PRRSV in lymphoid tissues for several months [39]. We hypothesize that PRRSV may interact with fDC in secondary lymph organs, such as the Med-LN, and this could impair fDC presentation of PRRSV antigens to B cells, which could contribute to the lack of production of neutralizing antibodies detected in this [10] and other PRRSV infection studies. PRRSV presence in follicles of Med-LN and the non-neutralizing Ab secretion at 35 dpi suggest a role of fDC in the immunopathogenesis of PRRSV.

The B and T cell depletion observed in F and IF areas of Med-LN during this study are in accordance with previous reports that showed B and T cell depletion in cortical areas of secondary lymph organs such as the thymus [44] and lymph nodes [45] of PRRSV infected pigs. The immune cell depletion in lymphoid tissues has recently been associated to apoptotic cell death indirectly induced in bystander cells by PRRSV infected cells [46]. We showed a T cell increase in the F, mostly at 7 dpi, that could be related to the migration of CD4^+^ helper T cells to the F area to assist the induction of B cell responses. During this study the macrophage population increased in the Med-LN, especially for the SU1-Bel group, which indicates that the SU1-Bel strain induces a higher inflammatory response in the lung than the other PRRSV-1 strains tested, in accordance with previous reports [9,11].

Production of type I IFNs (IFN-α/β) is critical to activation of the innate immune response against viral infection, as well as regulation of the induction of the adaptive immune response [47,48]. We detected either no changes or lower IFN-α transcript levels for all PRRSV inoculated groups, most noticeably at 7 dpi, which likely plays a role in delaying the onset of an effective innate immune response [14,49]. We showed that PRRSV can reduce the transcription of TNF-α which would also impair innate immune response and to delay the adaptive immune responses [50,51]. Interestingly, the IFN-α and TNF-α gene expression were positively correlated in LV infected pigs, both LV and SU1-Bel RNA levels were negatively correlated with the IFN-α gene expression, and SU1-Bel RNA levels were negatively correlated with the TNF-α gene expression. These results suggest that PRRSV downregulates these cytokine gene inductions at the site of infection, in agreement with previous studies [52]. These results also support the hypothesis that PRRSV-1 subtype 3 strains have developed the most efficient strategies to avoid the host immune responses among the PRRSV-1 genotype viruses. The upregulation of IFN-γ expression at 7 dpi is evidence of a delayed adaptive immune response in all PRRSV inoculated groups, as it has been previously described [53,54]. During this study a positive correlation between IFN-γ gene expression and PRRSV LV and 215–06 strains suggests that PRRSV-1 subtype 1 induction of IFN-γ expression depends directly on the presence of virus. It has been described that SOCS proteins are a pivotal regulator in both innate and adaptive immune responses [55]. Specifically SOCS1 acts as a negative regulator of IFN-γ signaling, inhibiting the activation of STAT1 and thereby the expression of IFN-γ mediated genes [56]. SOCS1 induction in PRRSV vaccinated/infected pigs has been described [57,58], which suggests SOCS1 expression in infected cells may be a mechanism to evade the host immune response. Nevertheless, the positively correlation of SOCS1 and IFN-γ gene expression in the LV group could be a result of its negative feedback control system. Additional in vitro experiments may be used to elucidate the proposed SOCS1 induction by PRRSV during the initial phase of virus infection.

The IL-23 protein is an IL-12p40-IL-23p19 hetero-dimer that is secreted by antigen presenting cells such as macrophages and DCs. This cytokine is necessary for the differentiation and survival of Th17 cells, which can induce a pro-inflammatory reaction and the secretion of TGF-β [59]. In this study we did not detect IL-12p40 transcripts; this could be explained by the transcription kinetics of this cytokine being earlier or later compared with the tissue sample collection points. The IL-23p19 is a limiting factor to produce a biologically active IL-23 protein, as IL-23p19 is not secreted in the absence of the IL-12p40 chain [60] and the IL-23p19 induction is typically lower than that of IL-12p40 [61]. In the present study, IL-23p19 was upregulated from 7 dpi, and this was statistically significant at 35 dpi in the F for all PRRSV groups, which suggest that PRRSV may modulate the cytokine environment to drive the differentiation of Th17 as opposed to antiviral Th1 cells.

The IL-10 transcription levels showed considerable variation between PRRSV strains as previously described [62]. Therefore, this study supports the idea that the induction of IL-10 as a mechanism to delay the host immune response is not a common strategy among the PRRSV-1 genotype. The TGF-β gene expression was either unaffected or downregulated in agreement with previous findings [63], and this may explain why a positive correlation between TGF-β and other cytokines were detected in this study.

The standard techniques used to take samples for mRNA extraction cannot select different tissue structures in the way that LMC can. We therefore conclude that LMC in combination with RT-qPCR is a powerful tool to enable the differentiation of transcriptomic profiles between different Med-LN compartments of PRRSV-1 infected pigs. In this study we show several examples that demonstrate how the immunopathogenesis of PRRSV-1 infection is associated with the site of infection; for instance, PRRSV-1 SU1-Bel showed a higher tissue pathogenesis and virulence compared with PRRSV-1 subtype 1 strains. We have also provide further evidence that PRRSV-1 strains avoid the innate immune response in infected pigs through the downregulation of IFN-α and TNF-α in F and IF areas of Med-LN, and inducing a T and B cell depletion in the cortex of Med-LN.
